# Serological Survey and Molecular Characterization of *Theileria annulata* in Sicilian Cattle

**DOI:** 10.3390/pathogens10020101

**Published:** 2021-01-21

**Authors:** Valeria Gargano, Valeria Blanda, Delia Gambino, Francesco La Russa, Sophia Di Cataldo, Antonino Gentile, Giorgia Schirò, Alessandra Torina, Javier Millán, Domenico Vicari

**Affiliations:** 1 Istituto Zooprofilattico Sperimentale della Sicilia “A. Mirri”, Via Gino Marinuzzi n. 3, 90129 Palermo, Italy; valeria.gargano@izssicilia.it (V.G.); francesco.larussa@izssicilia.it (F.L.R.); antogentile1980@gmail.com (A.G.); giorgia.schiro91@gmail.com (G.S.); alessandra.torina@izssicilia.it (A.T.); domenico.vicari@izssicilia.it (D.V.); 2 Programa de Doctorado en Medicina de la Conservación, Facultad de Ciencias de la Vida, Universidad Andres Bello, Santiago, Chile; m.dicataldohanna@uandresbello.edu; 3 Instituto Agroalimentario de Aragón-IA2 (Universidad de Zaragoza-CITA), 50013 Zaragoza, Spain; syngamustrachea@hotmail.com; 4 Fundación ARAID, 50018 Zaragoza, Spain; 5 Facultad de Ciencias de la Vida, Universidad Andres Bello, Santiago, Chile

**Keywords:** Piroplasmida, phylogenetic analysis, tick-borne pathogens, 18S rRNA, Sicily

## Abstract

Tropical theileriosis is a tick-borne disease caused by hemoprotozoan parasites with considerable veterinary and economic impact worldwide. Ticks transmitting the disease belong to the *Haemaphysalis*, *Rhipicephalus*, and *Hyalomma* genera. The *Hyalomma* genus is very common in Sicily (Italy) and represents the main *Theileria annulata* vector in the island. Data concerning the molecular epidemiology of this pathogen are missing in the region. In 2018–2019, blood and serum samples were collected from 480 cows in seven Sicilian farms from four different provinces. Seroprevalence in the farms ranged from 22% to 71%. Three farms were selected for molecular analysis consisting of real-time PCR targeting the almost complete 18S ribosomal RNA (rRNA). Four amplicons per farm were sequenced and phylogenetic analyses were carried out. The four sequences were identical within each farm and showed 92–99% identity with the other farms and with sequences from Genbank. According to the phylogenetic analysis, these three sequences and an additional one from a laboratory-cultured *Theileria annulata* strain obtained in 1999 belonged to a single *T. annulata* clade with good bootstrap support with other sequences from Italy, India, and Iran, indicating limited geographical and temporal genetic variability of the parasite. This study represents the first phylogenetic analysis of *T. annulata* in Sicily, which will be useful to improve the strategies for theileriosis control and prevention.

## 1. Introduction

Parasites of the *Theileria* genus (order Piroplasmida, family Theileridae) infect a wide spectrum of domestic and wild animals and are transmitted by Ixodidae ticks belonging to the *Haemaphysalis*, *Hyalomma*, and *Rhipicephalus* genera [1]. In recent years, there has been a growing interest in the diseases caused by *Theileria* spp., also due to the increased accuracy of the diagnostic tests [2]. Tropical theileriosis in cattle is caused by *Theileria annulata*, and it is characterized by leukocyte proliferation following pathogen infection [3]. Other symptoms include fever, lymphadenopathy, and anemia. If not treated, animals die within 3–4 weeks of infection [4]. This pathology also causes a significant reduction in animal fertility. In Sicily, theileriosis is one of the most common tick-borne diseases with several outbreaks noticed each year [5]. The current control measures include the use of acaricides for vector control, therapy with buparvaquone, and vaccination [6]. Several pathogen molecules able to induce the host immune responses are currently under investigation as candidate vaccine antigens [7]. In Italy, vaccine prophylaxis against tropical theileriosis is not carried out since the currently available live-attenuated vaccine is not commercialized in Europe [8]. The success of the therapy depends on the timeliness of the execution of the pharmacological treatment [9]. For this reason, molecular biology represents a very efficient diagnostic approach to contrast the disease, since molecular methods, such as real-time PCR, allow rapidly detecting the pathogen and promptly initiating control actions [10].

Moreover, the advances in sequence data analysis allowed an improvement in pathogen identification and characterization. Ribosomal RNA (rRNA) is the most used genetic marker for phylogenetic analysis in eukaryotic organisms due to the conserved function and structure of the 18S rRNA molecule. This gene has been sequenced from several organisms, resulting in a large database for sequence comparisons.

The marker 18S rRNA gene is widely used for determining evolutionary patterns and similarity among the *T. annulata* species due to the presence of a conserved and hypervariable region (V4). Indeed, the molecule also possesses phylogenetically informative variable regions that are useful for determining relationships among strains [11].

*T. annulata* remains one of the most common tick-borne pathogens affecting domestic ruminants not only in Italy [5] but in several countries of Europe [12,13]. In Sicily, *Hyalomma lusitanicum* has been proposed as the main *T. annulata* vector in cattle, and this tick species is relatively prevalent in extensive cattle breeding [14,15]. Although the presence of *T. annulata* in Sicily is well known, phylogenetic studies concerning *T. annulata* have been carried out in other European countries but not in Italy [16,17,18].

This study was aimed at the phylogenetic characterization of *T. annulata* strains present in cattle in Sicily to better understand the epidemiology of the parasite on the island. 

## 2. Results

Overall, serological prevalence in the Sicilian cattle was 26.0% (95% confidence intervals = 31.7–41.5). At the farm level, observed prevalence values ranged from 22% to 71% (Table 1).

According to the serological results, the three farms with the highest prevalence values and showing at least 10 animals serologically positive for *T. annulata* were selected for molecular studies (Farms 3, 5, and 6; Table 1).

For each of the three farms, four animals were selected for the sequencing of the almost complete 18S rRNA gene. Sequences obtained from the animals of the same farm were identical to each other and were considered as a single oligonucleotide sequence (Sequences 2–4; Table 2). These three sequences showed 98–100% identity.

**Figure 1:**
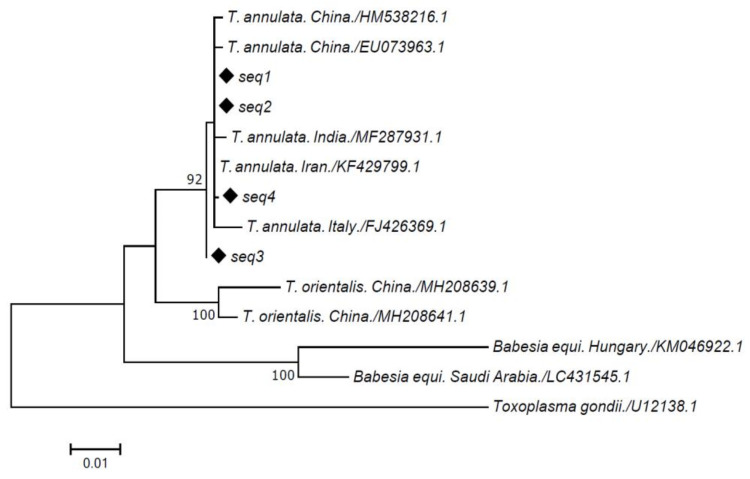
Phylogenetic tree obtained in this study using the 18S ribosomal RNA (rRNA) gene sequences.

Moreover, a further 18S rRNA sequence, named sequence 1 (Seq1), was obtained from the *T. annulata* strain, isolated several years earlier at our institute and maintained in culture as described above. The four obtained sequences shared between 99.8% and 100% nucleotide sequence identity with sequences previously reported in GenBank (AY508463 and MN944852, respectively). Alignments of multiple 18S rRNA gene sequences revealed four haplotypes of *T. annulata* rRNA. The phylogenetic analysis (Figure 1) indicated that our sequences were classified in a single *T. annulata* clade with good bootstrap support with other sequences from Italy (FJ426369) and India and Iran (MF287931 and KF429799). Only sequence 3 rooted apart from the other sequences, indicating a likely different origin from the others. This finding calls for further investigation on the particularities of this farm.

## 3. Discussion

*T. annulata* remains one of the most common tick-borne pathogens affecting domestic ruminants in several European countries [5,12,13]. The observed seroprevalences are lower than those reported in other Mediterranean countries such as Tunisia or in other countries such as Sudan or Iraq [18,19,20,21,22]. Notwithstanding this, the parasite is actively circulating in the Sicilian cattle farms. In this study, the 18S rRNA gene partial sequences were used to evaluate the phylogenetic relationships of Sicilian *T. annulata* strains with other *T. annulata* strains, as well as with different *Theileria* species. Although the presence of *T. annulata* in Sicily is well known, phylogenetic studies concerning *T. annulata* have been carried out in other European countries but not in Italy [14,15,16,17,18].

In this study, Sicilian sequences formed a unique clade close to sequences from Italy, India, and Iran, confirming a molecular study conducted on a vaccine strain in Iran [23]. In that study, the 18S rRNA gene sequence of the *T. annulata* Iran vaccine strain showed from 97.9% to 99.9% identity with strains from China, Spain, and Italy. Moreover, a previous study concerning *Theileria* species in small ruminants in Italy also reported a closer association between the Italian *Theileria ovis* strain and *Theileria* spp. from Namibia and Iran [11]. These results may be the consequence of movements of animals and ticks between these countries, a hypothesis further strengthened by the usual practice of ancient peoples of migrating from these areas in the past, bringing with them some farm animals [24]. Furthermore, our data show that the sequences obtained from field samples are not substantially different from the sequence obtained from the strain isolated almost 20 years ago and cultured in our laboratory, suggesting a low rate of genetic variability of the pathogens.

## 4. Materials and Methods

### 4.1. Field Sample Collection

From September 2018 to March 2019, seven farms were investigated for *T. annulata* presence. Four farms were in the province of Ragusa, one in the province of Syracuse, and one each in the provinces of Enna and Palermo. All the selected farms were directed to the production of milk and meat. Animals of each farm, according to the regulations in force, are housed and cannot come into contact with the animals of other farms. Ethylenediaminetetraacetic acid (EDTA)-treated and untreated blood samples were obtained from each animal. The serum was separated by centrifugation and stored with EDTA-treated blood samples at −20° C.

### 4.2. In Vitro Cultivation of T. annulata Schizonts

Peripheral blood mononuclear cells (PBMCs) were isolated from heparinized blood samples as described elsewhere [25]. *T. annulata* schizonts from a positive blood sample obtained in the year 1999 were isolated and maintained in our laboratory in Roswell Park Memorial Institute (RPMI) 1640 medium added with 2 mM l-glutamine, 1% penicillin/streptomycin, and 10–20% fetal bovine serum (pH at 7.3) and incubated at 37° C in 5% CO_2_. Once the cell concentration of 5–9 × 10^5^ cells/mL was reached, the culture was propagated in fresh soil.

### 4.3. Serological Analysis

Samples taken from animals housed on the seven farms were subjected to serological analysis. The presence of antibodies against *T. annulata* was assessed by immunofluorescence using a protocol modified from Burridge and Kimber [26].

### 4.4. Molecular Analysis and Phylogenetic Studies

For molecular analysis, samples belonging to the three farms with the highest prevalence values and showing at least 10 animals serologically positive for *T. annulata* were selected.

DNA was extracted using the PureLink Genomic DNA kit (Thermo Fisher Scientific, Waltham, MA, USA) according to the manufacturer’s instructions. DNA was extracted from the blood of field samples and PBMCs infected by *T. annulata*, and 200 ng of each purified genomic DNA was analyzed on 1% agarose gel (data not shown). Furthermore, the A206/A280 ratio was evaluated to verify the quality of the extracted DNA. Each sample was found suitable for further analysis with an A206/A280 ratio close to 2.0. *T. annulata* DNA was detected through real-time PCR [27]. For each positive farm, four samples, with a Ct value below 28, were selected to carry out the phylogenetic analysis.

### 4.5. Phylogenetic Study

#### 4.5.1. 18S rRNA Gene Amplification and Sequencing

A portion of the 18S rRNA gene was obtained with specific primers 18S TBF and 18S TBR as described elsewhere [28]. The amplified product of approximately 1500 bp was used for sequencing analysis. DNA sequences were determined using the dideoxy chain termination method with a commercial DNA sequencing kit (BigDye™ Terminator v3.1 Cycle Sequencing Kit, Applied Biosystems™ (Thermo Fisher Scientific, Waltham, MA, USA) according to the manufacturer’s instructions. The obtained sequences were analyzed for nucleotide sequence identity by comparing them with reference strains in the GenBank database using the Basic Local Alignment Search Tool (BLAST). Multiple sequence alignments were performed using the ClustalW algorithm. Similarity matrices were then constructed from aligned sequence data by single distance using the Jukes and Cantor or the Kimura two-parameter model [29]. Phylogenetic trees were constructed using the neighbor-joining and the maximum parsimony methods as implemented in the Mega 2.1 package, deleting all the gap sites. *Toxoplasma gondii* was included as an outgroup [30]. The stability or accuracy of inferred topologies was assessed via bootstrap analysis of 1000 replicates. 

#### 4.5.2. Sequence Accession Numbers

The GenBank accession numbers for the gene sequences obtained in this study are MN944852, MT341815, MT341857, and MT341858.

## 5. Conclusions

As for other tick-borne pathogens, the distribution of *Theileria* spp. infections is related to several factors, including the presence of the tick vector species, the host, and the reservoir. Our data showed the presence of *T. annulata* in sampled animals, confirming that this parasite is prevalent in intensive cattle breeding.

Furthermore, our data show that the sequences obtained from field samples are not substantially different from the sequence obtained from the strain isolated almost 20 years ago and cultured in our laboratory, suggesting a low rate of genetic variability of the pathogen.

Serological investigations and characterization of *T. annulata* strains circulating in an area are useful to identify possible routes of entry of new strains into a territory, as well as identify the most appropriate therapeutic approaches, including vaccination strategies, for the prevention and control of the pathogen.

## Figures and Tables

**Table 1:** Results of serological analysis obtained in this study. ID, identifier.

Farm ID	Province	Sampled Animals	Positive Animals	Prevalence
1	Ragusa	216	48	22%
2	Ragusa	53	52	23%
3	Siracusa *	101	51	50%
4	Ragusa	49	18	37%
5	Ragusa *	21	15	71%
6	Enna *	17	10	59%
7	Palermo	24	8	33%

* Farms selected for the molecular study.

**Table 2:** Details of *Theileria annulata* sequences obtained in this study.

Sequence ID	GenBank Accession Number (This Study)	Provenience	Province	Number of Sequences from Positive Animals	GenBank Accession Number of the Closest Sequence
Sequence 1	MN944852	Cultured Strain	Palermo (Sicily)	1	KF429799
Sequence 2	MT341815	Field Strain	Ragusa (Sicily)	4	MK849885
Sequence 3	MT341857	Field Strain	Syracuse (Sicily)	4	KT367871
Sequence 4	MT341858	Field Strain	Enna (Sicily)	4	KT367868

## Data Availability

10–20% GenBank accession numbers of the sequences obtained in this study are MN944852, MT341815, MT341857, and MT341858, available at https://www.ncbi.nlm.nih.gov/genbank/.
